# A Small Peptide with Potential Ability to Promote Wound Healing

**DOI:** 10.1371/journal.pone.0092082

**Published:** 2014-03-19

**Authors:** Jing Tang, Han Liu, Chen Gao, Lixian Mu, Shilong Yang, Mingqiang Rong, Zhiye Zhang, Jie Liu, Qiang Ding, Ren Lai

**Affiliations:** 1 Key Laboratory of Animal Models and Human Disease Mechanisms of Chinese Academy of Sciences and Yunnan Province, Kunming Institute of Zoology, Kunming, Yunnan, China; 2 Department of Breast Surgery, the First Affiliated Hospital of Nanjing Medical University, Nanjing, Jiangsu, China; 3 College of Veterinary Medicine of Jiangsu Animal Husbandry and Veterinary College, Taizhou, Jiangsu, China; 4 Graduate University of Chinese Academy of Sciences, Beijing, China; Institut national de la santé et de la recherche médicale - Institut Cochin, France

## Abstract

Wound-healing represents a major health burden, such as diabetes-induced skin ulcers and burning. Many works are being tried to find ideal clinical wound-healing biomaterials. Especially, small molecules with low cost and function to promote production of endogenous wound healing agents (i.e. transforming growth factor beta, TGF-β) are excellent candidates. In this study, a small peptide (tiger17, c[WCKPKPKPRCH-NH_2_]) containing only 11 amino acid residues was designed and proved to be a potent wound healer. It showed strong wound healing-promoting activity in a murine model of full thickness dermal wound. Tiger17 exerted significant effects on three stages of wound healing progresses including (1) the induction of macrophages recruitment to wound site at inflammatory reaction stage; (2) the promotion of the migration and proliferation both keratinocytes and fibroblasts, leading to reepithelialization and granulation tissue formation; and (3) tissue remodeling phase, by promoting the release of transforming TGF-β1 and interleukin 6 (IL-6) in murine macrophages and activating mitogen-activated protein kinases (MAPK) signaling pathways. Considering its easy production, store and transfer and function to promote production of endogenous wound healing agents (TGF-β), tiger17 might be an exciting biomaterial or template for the development of novel wound-healing agents.

## Introduction

The skin is the largest organ and its primary function is to serve as a protective barrier against outside environment and excessive water loss. Skin consists of two tissue layers: a keratinized stratified epidermis and an underlying thick layer of collagen-rich dermal connective tissue providing support and nourishment [1], [2]. Cutaneous injury initiates a complex set of events which finally lead to wound repair. Healing of skin wounds progresses is a dynamic process including three highly integrated and overlapping stages: (1) inflammatory reaction stage consisting of the extravasation of blood constituents with resultant platelet aggregation, blood coagulation and migration of inflammatory cells to the wound site; (2) proliferative phase involving the migration and proliferation of keratinocytes, fibroblasts and endothelial cells, leading to reepithelialization and granulation tissue formation; and (3) tissue remodeling phase restoring tissue structural integrity and functional competence [3]. Lots of cellular and molecular biological studies demonstrated that many cytokines, growth factors, and proteases are closely involved in the wound healing process to complete normal tissue repair. Besides providing a structural barrier, the skin contains several immune cells that can be activated by invading pathogens or skin damage. One of the most important immune cells involved in wound healing is the macrophage, which exhibits different immunological functions in the skin, including phagocytosis and antigen-presentation [4]. Furthermore, they can produce many cytokines and chemokines to orchestrate the wound healing process throughout the different phases [5], [6].

Two antimicrobial peptides (tigerinin-RC1: RVCSAIPLPICH; tigerinin-RC2: RVCMAIPLPLCH) have been identified from the skin secretions of *Fejervarya cancrivora* in our previous work [7]. They have been found to exert wound-healing activity (Data not shown). In this work, we have synthesized several peptides based on tigerinins to screen candidates containing potential wound-healing ability. Among these peptides, a small peptide, named tiger17, was manifested to be a potent healer of skin wound.

## Materials and Methods

### Peptide Synthesis

Tiger17 (c[WCKPKPKPRCH-NH_2_]) was synthesized by solid phase synthesis on an Applied Biosystems model 433A peptide synthesizer by GL Biochem (Shanghai) Ltd. (Shanghai, China) and analyzed by HPLC and mass spectrometry to confirmed purity higher than 98%. Molecular weight of tiger 17 was 1376.79 Da determined by MALDI-TOF mass spectral analysis, which indicated that the peptide was in cyclic form.

### Cell Lines and Animals

Both immortalized human keratinocyte (HaCaT) cell line and human skin fibroblasts (HSFs) cell line were obtained from Kunming Cell Bank, Kunming Institute of Zoology, Chinese Academy of Sciences. The cell lines have been checked free of mycoplasma contamination by fluorescent staining. The intraspecies determination has been performed by STR analysis before providing it to the user. DNA profile is consistent with other DNA profiles on records for these cell lines. All the cell lines in this work have been commonly used in our institution, and are not de novo cell lines [8].

Male Kunming mice between the weight ranges of 25–30 g were used in the study. The mice were maintained under standard acclimatization conditions of 12 h light/dark cycle at 25°C and were provided standard rodent feed procured. All surgery was performed under sodium pentobarbital anesthesia, and all efforts were made to minimize suffering. All the experimental protocols to use cell lines and animals were approved by the Animal Care and Use Committee at Kunming Institute of Zoology, Chinese Academy of Sciences (Permit Number: 33-2397).

### Cell Proliferation Assay

HaCaT cells and HSFs were cultured in DMEM (Invitrogen) supplemented with 10% fetal bovine serum, 100 units/ml of penicillin, and 100 units/ml of streptomycin in a humidified 5% CO_2_ atmosphere at 37°C, respectively. HaCat and HSF cells (2×10^4^ per well, 180 μl) were plated into a 96-well plate, respectively. After an overnight incubation, both HaCat and HSF cells were adhered to the plate, and then 20 μl of tested samples dissolved in DMEM with different concentration (2.5, 5, 10, 20 μg/ml) were added to the wells for 24 h incubation. The same volume of DMEM was used as blank control. At the end of the incubation, 20 μl of 3-(4-5-di-methylthiazol-2-yl)-2,5diphenyltetrazoliumbromide solution (MTT, 5 mg/ml) was added to each well, which turns into a purple formazan product in the presence of viable mitochondria in living cells, and the cells were further incubated for 4 h at 37°C. The cells were solved in 150 μl of DMSO, and the absorbance of formazan at 570 nm was measured in an ELISA reader. The optical density reflects the level of cell metabolic activity. Experiments were repeated independently in triplicate.

### Wound Healing Scratched Assay

HaCat keratinocyte cells (1×10^6^) were seeded in a 6-well plate and cultured as monolayer to confluence overnight prior to serum starvation for 24 h. The monolayer was then scratched with a yellow 200 μl pipette tip to create an approximate 1-mm-wide wound area, and washed twice with PBS to remove floating cells. After the line scratch, 2 ml DMEM was added into every well. To observe the effect of tiger17 on keratinocytes migration, cells were incubated with tiger 17 (20 μg/ml) for various time periods (from 0 h to 36 h), in the presence of mitomycin C (5 μg/ml) to prevent cell proliferation. Images of the wounded cell monolayer were taken using a microscope (Olympus, Tokyo, Japan) at 0, 24, and 36 h after scratched wounding. Cell migration activity was expressed as the percentage of the gap relative to the total area of the cell-free region immediately after the scratch, named the repair rate of scarification, using Image J software (National Institutes of Health, Bethesda, MD, USA). For each plate, 6 randomly selected images were acquired. All experiments were independently carried out in triplicate.

### Experimental Excision Wound Creation

The animals were anesthetized with intraperitoneal injection of 100 μl solution containing 1% pentobarbital sodium (0.1 ml/20 g body weight). The dorsal hair of the mouse was shaved and disinfected with 70% ethanol swab to prepare the back skin for generation of a standardized full-thickness cutaneous wound. Two 6×6 mm full-thickness excisional wounds were created on the back of each mouse. At the end of the surgical procedure, cages were placed near to a heating apparatus until mice fully recovered from anesthesia.

### Animal Grouping and Treatment Schedule

Mice bearing full-thickness wound were randomly divided into two groups of 10 animals each, named T group and E group. T group was separately provided 20 μl (20 μg/ml) tiger17 or equal vehicle on the two wound of a mouse; and for E group, 20 μl tiger17 (20 μg/ml) or EGF (100 μg/ml) solution was respectively applied topically on the two incisions of a mouse. Some mice in each group were euthanized on days 3, 7, 8 and 9 post-wounding, and skin tissue samples from the wound site were collected from all of the mice for histological and immunohistochemical analysis.

### Measurement of Wound Healing Rate

Wound closure was documented with a digital camera. The residual wound areas (percentage of wound areas to initial ones) were calculated from the photographs using an image analysis program (Image J, NIH) by tracing the wound margin and calculating the pixel area. The measurements were performed in triplicate and mean values of consecutive tracings were computed and expressed as percentage of closure from the original wound. The Residual Wound Area (%) = [R_(2∼9)_/R_(0)_]×100, where R_(0)_ and R_(2∼9)_ denote the remaining area at the same day of operation and postoperative days 2∼9, respectively. The wound-healing curve was obtained using Graphpad Prism version 5.

### Histology and Immunohistochemistry

Wound specimens, including a 5 mm margin of non-wounded skin from the wound site of the animals after treatment with tiger17 and of untreated controls were removed after sacrifice and fixed in 10% formalin, dehydrated through a graded series of ethanol, cleared in xylene, and embedded in paraffin wax. 5 μm thick sections were cut and stained with Hematoxylin and Eosin (H&E) for histological analysis. Histologic sections and IPLab imaging software (BD Biosciences, Bedford, MA) were used to measure the re-epithelialization capability of the wound. Neo-epithelial formation rate was determined by measuring the lengths of the tongues of neo-epithelium migrating from either side of the wound over the wound bed from the zone of proliferation at the margin of the uninjured and wounded skin. This was compared to the length of the total wound to generate the “percent reepithelialized”. The reepithelialized % = neoepithelial tongue+neoepithelial tongue/total wound length×100%.

For immunohistochemistry, 3 μm sections were incubated with anti-F4/80 primary antibody (1∶200 dilution, ab111101, abcam), anti-TGF-β1 primary antibody (1∶500, ab92486, abcam), or anti-α SMA primary antibody (1∶1000 dilution, ab32575, abcam) by an overnight incubation at room temperature after blocking endogenous peroxidase and nonspecific binding. Control sections were incubated in parallel in the same dilution buffer without the primary antibodies. After washing in PBS, the sections were exposed to biotinylated goat antirabbit IgG (1∶200 dilution, ab150077, abcam) for 1 h at room temperature. The avidin-biotinylated horseradish peroxidase complex (ABC) solution was prepared and then incubated with the sections. Immunoreactivity was visualized by incubation with H_2_O_2_ (0.01%) and DAB·4 HCl (0.05%) that under nickel intensification gave rise to a dark brown deposit at the site of antigen binding. Sections were air-dried, dehydrated in ethanol, cleared in xylene and coverslipped using DPX mounting medium.

### ELISA

Macrophage RAW264.7 cells (1×10^6^/well) were plated and adhered to a 96-well culture plate. After 16 h incubation with tiger17 (2.5, 5, 10 and 20 μg/ml) or vehicle, the supernatants were respectively collected by centrifugation (12000 rpm) for 30 minutes at 4°C. Supernatants were stored at −20°C until use. Cytokines released in the cell-free supernatants from non-stimulated or stimulated culture macrophage cells were measured with ELISA kits from R&D Systems (Minneapolis, MN). ELISA was performed according to the manufacturer’s instructions. Experiments were independently carried out in triplicate.

### Western Blot Analysis

Raw 264.7 murine macrophage cells (1×10^6^/well) were plated and adhered to a 24-well culture plate. The cells were then transferred to serum-free DMEM for 16 h incubation. The cells were incubated with various concentrations of tiger17 (2.5, 5, 10 and 20 μg/ml) or blank, 1 h incubation for MAPKs pathway, and 18 h incubation for Smads pathway. After incubation, the cells were collected by centrifugation (800 rpm, 5 min) and washed twice with ice-cold PBS. The washed cell pellets were resuspended in 150 μl of extraction lysis buffer (50 mM Tris-HCl, pH 7.4; 1% Nonidet P-40; 0.25% sodium deoxycholate; 150 mM NaCl; 1 mM EDTA; 1 mM phenylmethylsulfonyl fluoride; 1 μg/ml each of aprotinin, leupeptin, and pepstatin; 1 mM sodium orthovanadate; and 1 mM NaF) and incubated for 30 min at 4°C. Cell debris was removed by centrifugation (12000 rpm, 30 min, 4°C), followed by quick freezing of the supernatants. The protein concentration was determined using the Bradford protein assay. Forty micrograms of cellular protein from treated and untreated cell extracts were separated on a 12% SDS-PAGE gel and electroblotted onto a polyvinylidene difluoride membrane. The immunoblot was incubated with blocking solution (5% skim milk) at room temperature for 1 h, followed by incubation overnight with a primary antibody against the phosphorylated and total forms of Erk1/2, SAPK/JNK, p38 MAPK, Smad2 or Smad3 at 4°C, respectively. For Smad7, only a primary antibody detected the total form expression of Smad7. The blots were washed three times with Tween 20/Tris-buffered saline (TBST) and incubated with a 1∶1000 dilution of horseradish peroxidase conjugated secondary antibody for 1 h at room temperature. The blots were again washed three times with TBST and then developed by enhanced chemiluminescence (Tiangen Biotech). Experiments were independently carried out in triplicate.

### Statistical Analysis

Statistical analysis was performed by using the one-way analysis of variance test for the comparison of multiple variables, and Student’s test was used for comparing two variables. *P* value<0.05 was considered statistically significant. Results are shown as mean ± SEM. Graphs were generated by using GraphPad Prism version 5 (Graph Software, San Diego, CA).

## Results

### Tiger17 Enhanced Cell Proliferation and Cell Migration

**Figure 1:**
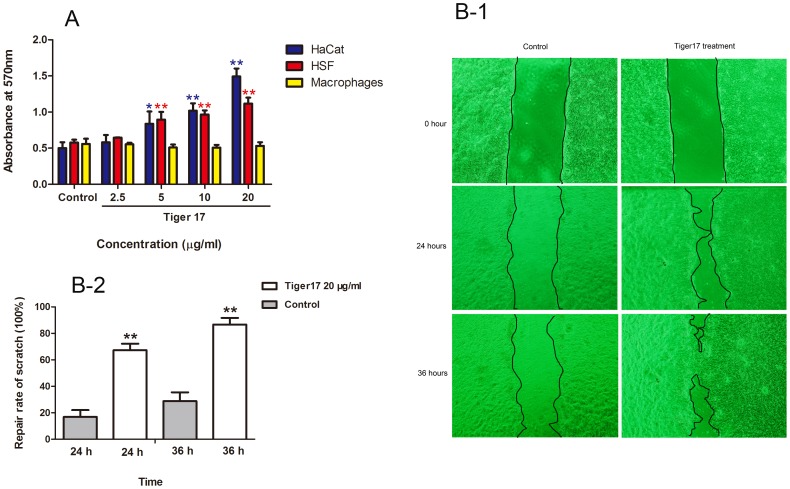
Tiger17 promotes HaCat cells proliferation and migration. (A) Keratinocytes, fibroblasts or macrophages (2×10^4^ cells/ml) were separately cultured in wells of 96-well plate, and cells were incubated with various concentration tiger17 (2.5, 5, 10, 20 μg/ml) or sterile H_2_O alone for 24 h. After incubation with 5 mg/ml MTT for 4 h, absorbance was determined. It was very obviously that both HaCat keratinocytes and HSFs were increased by tiger17 treated, in a concentration dependent manner. Tiger17 had little effect on macrophages proliferation. Values are the mean ± SE of three independent experiments. *P<0.05, **P<0.01 as compared between tiger17-stimulated and non-stimulated cells. (B) HaCat keratinocytes cells (1×10^6^ cells/ml) were cultured in wells of 6-well plate; cells were scratched and incubated with tiger17 (20 μg/ml) or vehicle. (B-1) Cells migration was recorded by photomicrograph at post-scratched 0 hour, 24th hour and 36th hour. The black line denoted the margin of gap. (B-2) The repair rate of scarification was calculated by Image J. According to the formula, repair rate of scarification % = (the gap width of 0 hour - temporal gap width)/the gap width of 0 hour ×100%. Scarification width was measured in triplicates wells. The distance of matched 6 pairs of points was separately measured in each well for averaging the gap width. Values are the mean ± SE of three independent experiments. **P<0.01 as compared between Tiger17-treated and untreated cells.

Keratinocytes and fibroblasts are important cells that dominantly take part in proliferation phase of wound healing [9]. As illustrated in Fig. 1A, tiger17 promoted HaCaT keratinocytes and human skin fibroblasts (HSFs) proliferation in a concentration-dependent manner. At the concentration of 2.5, 5, 10 and 20 μg/ml, tiger 17 increased 20, 60, 110 and 200% HaCaT keratinocyte proliferation while the corresponding HSF proliferation increase was 15, 40, 60 and 95%, respectively. However, tiger17 exerted little effect on the proliferation of Raw 264.7 murine macrophage (as shown in Fig. 1A).

**Figure 2:**
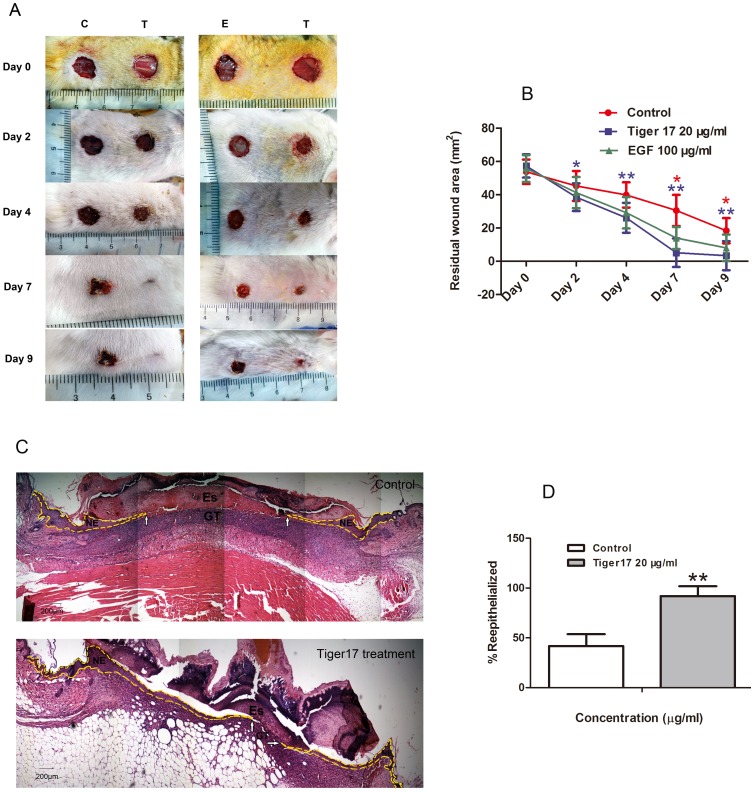
Topical application of tiger17 accelerated healing of full-thickness skin wounds in mice. Two full-thickness wounds were made on the back of a mouse. Mice bearing wound were randomly divided into two groups, T group and E group, 10 mice each. T group was separately provided 20 μl of tiger17 (20 μg/ml) or vehicle; and E group was respectively applied 20 μl of tiger17 (20 μg/ml) or 20 μl of EGF (100 μg/ml) solution. (A) Images of a representative mouse from each group taken on post-operative day 0, 2, 4, 7 and 9 were shown. (B) Evaluation of wound closure by morphometrical analysis of wound areas (Image J, NIH). Wound residual area was calculated (n = 10) from three independent experiments. *P<0.05; **P<0.01 (red asterisk represented tiger 17 treatment vs EGF treatment, and blue asterisk represented tiger 17 treatment vs control). (C) Post-operative day 8, healing skin was examined by H&E staining sections. The gap between neo-epithelial tongue was much narrower in tiger17-treated group (white arrows pointed) than control (white arrows pointed); and neo-epithelial tongue, marked by yellow dotted line, was much longer in the same magnitude of enlargement in tiger17 treatment mice. NE: neo-epithelium; GT: granulation tissue; Es: eschar. (D) Reepithelialization rate. Wound reepithelialization rate was calculated from the H&E stain sections. Bars = mean ± SEM. *P<0.05; **P<0.01 (tiger17 treatment vs control). Average and standard error of the mean were derived from three independent experiments and 6 different sections per experiment.

During the healing of cutaneous wound, except for cell proliferation, keratinocytes migration is also very important. Pioneer keratinocytes, which early migrate to wound area and form neo-epithelial tongue to cover the wound incision, consequently, in favor of proper and timely wound reparation [10]. An *in vitro* cells scratch assay was performed to investigate effect of tiger17 on keratinocytes migration. Keratinocytes migration rate was based on the efficiency of monolayer cells invading the wound region with the tiger17 treatment for 0 to 36 h. Tiger17 treatment significantly increased migration rate of keratinocytes into the wound area (Fig. 1B-1). As expected, the prewound region appeared narrower than the vehicle control, which had an obviously larger denuded area after tiger17 treatment for 24 h. After the treatment for 36 h, the tiger17-treated wound area was almost completely closed while the control still had a wide gap. The repair rate of scarification was calculated by Image J software, as shown Fig. 1B-2.

### Tiger17 Accelerated the Healing of Full-thickness Wounds in Mice

Since tiger17 induced the proliferation and migration of cultured keratinocytes *in vitro* (Fig. 1), it would be expected to accelerate wound healing *in vivo*. A full thickness skin wound healing model was used to assess the effects of tiger17 topical application on wound healing. Tiger17 or vehicle was topically painted to the wounds twice daily. Images of the wounds were acquired on the same day of operation and post-operative day 2, 4, 7 and 9, respectively. The residual wound area in tiger17-treated mice on post-injury day 2, 4, 7 and 9 was 67.3, 45.6, 8.9 and 5.9% while that in EGF-treated mice was 73.9, 52.3, 24.9 and 14.3%, in vehicle mice was 84.1, 74, 56.7 and 34.1%, respectively (Fig. 2A and B). No adverse effects on the body weight, general health or behavior of the mice were observed after topical tiger17 treatment (Data not shown).

### Tiger17 Promoted Re-epithelialization of Skin Wound

The effects of tiger17 on wound healing were further investigated by histological analysis. Tiger17-treated and control groups mice were euthanized at post-operative day 8. The wounds with a 0.5 cm unwounded skin border were collected. A histological evaluation of skin tissue sections stained with Hematoxylin and Eosin (H&E) was performed. This analysis model has been previously used to measure an important parameter of wound healing: the reepithelialization [11], [12]. The level of reepithelialization was determined by measuring the lengths of neo-epithelial tongue migrating from either side of the wound over the wound bed from the zone of proliferation at the margin of the uninjured and wounded skin [13]. Histological analysis revealed that tiger17-treated mice promoted regeneration of neo-epidermal tissues (neo-epithelial tongue) and restoration of dermal in the wound as illustrated in Fig. 2C. Neoepithelial tongue was nearly covered the whole wounded area and granulation tissue in the tiger17-treated mice (reepithelialization 92%, Fig. 2D). In contrast, only partial neoepithelial tongue was found in the control (reepithelialization 42%, Fig. 2D).

### Effects on Cytokine Secretion

**Figure 3:**
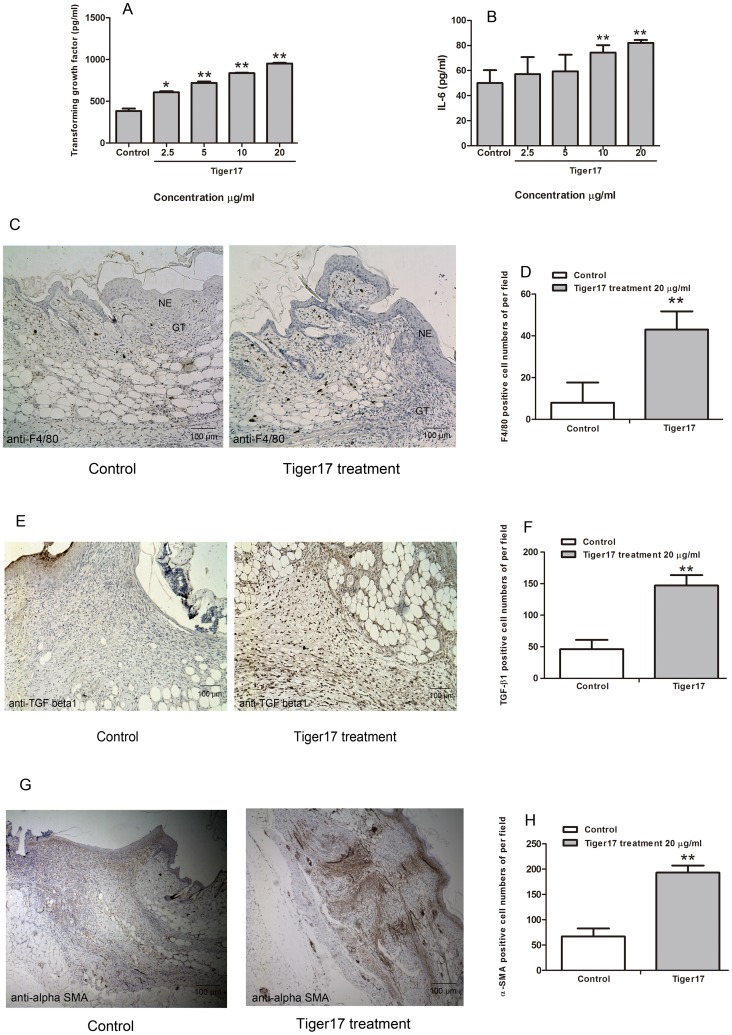
Tiger17 promoted macrophages recruitment, TGF-β1 upexpression and myofibroblasts differentiation. (A, B) Concentration of TGF-β1 or IL-6 (pg/ml) in the control and various concentration tiger17 (2.5, 5, 10, 20 μg/ml)-treated supernatant. Values are expressed as mean ± SE of triplicate, three independent experiments. *P<0.05; **P<0.01 (tiger17 treatment vs. control). (C, E, G) Immunohistochemical results for anti-F4/80, anti-TGF-β1 and anti-α-SMA. (D, F, H) F4/80, TGF-β1, or α-SMA positive cell numbers of per high power field were significant different between tiger17 treatment and control, **P<0.01. Average and standard error of the mean were derived from three independent experiments and 6 different fields each section (×100).

Many cytokines, such as TGF-β1, IL-1β, IL-6 and TNF-α have been a particular focus in recent research owing to their important roles in wound healing [14]–[16]. The effects of tiger17 on cytokines secretion in murine macrophages cell line RAW264.7 were tested using ELISA. TGF-β1 production was significantly increased in tiger17-stimulated supernatant compared with control (Fig. 3A). TGF-β1 concentration in supernatant was increased from 607.69 to 718.96 pg/ml (118.31%), 836.92 pg/ml (137.72%) and 951.92 pg/ml (156.65%) after the incubation of tiger17 at 2.5, 5, 10, and 20 μg/ml for 16 h, respectively (Fig. 3A). IL-6 secretion was also increased in a concentration dependent manner in tiger17-treated cells. Compared with the basic level of 51 pg/ml, 2.5, 5, 10, and 20 μg/ml tiger17 induced 58, 60, 73 and 81 pg/ml IL-6 secretion, respectively (Fig. 3B). Tiger17 had little effect on IL-1β and TNF-α secretion (data not shown).

### Tiger17 Induced Macrophage Recruitment, TGF-β1 Secretion and Fibroblast-to-myofibroblast Transition in Skin Wound

Macrophages take part in each phase of wound healing including host defence, the promotion and resolution of inflammation, the support of cell proliferation and tissue restoration following injury [17]. As illustrated in Fig. 3C, immunohistochemical analysis revealed that lots of macrophages were recruited to unwounded skin margin closed to neo-epidermis and neo-dermis in tiger17-treated skin wound. Compared ∼7 F4/80 (a specific marker of macrophage)-positive cells per field in vehicle-treated skin wound, there are ∼43 F4/80-positive cells per field in tiger17-treated skin wound (Fig. 3D).

TGF-β1 *in vivo* expression was also detected by immunohistochemical analysis as illustrated in Fig. 3E. Comparison with the vehicle group, TGF-β1 positive stain cells were much intensive in tiger17-treated group (Fig. 3E). There are ∼48 and 149 TGF-β1 positive stain cells per field in vehicle- and tiger17-treated group, respectively (Fig. 3F). Tiger17’s effect on TGF-β1 expression level *in vivo* was identical to that *in vitro* (Fig. 3A, E and F).

Macroscopic morphologic photographs and H&E sections revealed that wound contraction was significant in tiger17-treatment skin wound (Fig. 2A and C). Fibroblast-to-myofibroblast transition plays important role in cutaneous wound healing [18]. Myofibroblasts are activated fibroblasts, which have differentiated into a contractile phenotype and were characterized by the expression of a smooth muscle actin (α-SMA) [19]. Expression level of α-SMA, the marker of myofibroblast differentiation, was significantly increased by tiger17 treatment onto the wounds, as shown by immunohistochemical analysis (Fig. 3G). There were ∼60 and 195 α-SMA positive stain cells per field in the skin wound of vehicle- and tiger17-treated group on post-operative day 9, respectively (Fig. 3H).

### Effects of Tiger17 on TGF-β/Smad Signal Pathway

**Figure 4:**
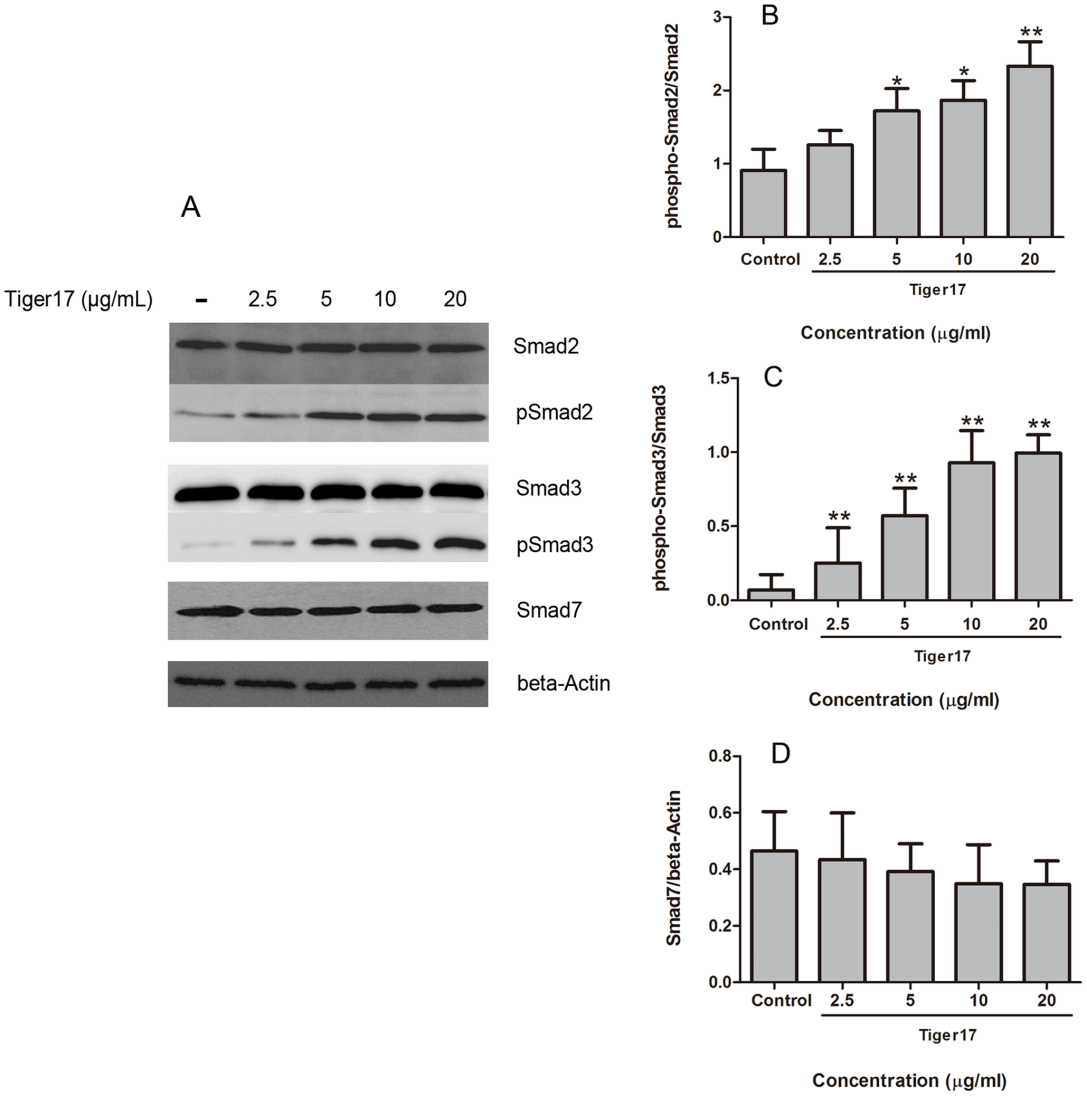
Effects of tiger17 on the Smads signal pathway. (A) Raw 264.7 cells were treated with the indicated concentrations of tiger17 (2.5, 5, 10, 20 μg/ml) or vehicle; Western blotted for total-Smad2, p-Smad2, total-Smad3, p-Smad3 or toal-Smad7 was performed. β-Actin was used as the internal control. (B, C, D) The band intensity of various inducer of Smads pathway = each phosphate-contents/homologous total-contents. The band intensity of inhibitory Smad7 = each Smad7-contents/the same concentration tiger17-stimulated β-Actin. (NIH, Image J). Values are the mean ± SE of three independent experiments, *P<0.05, **P<0.01.

Tiger17 has been demonstrated to induce TGF-β1 secretion *in vitro* and *in vivo* (Fig. 3A and E). The Smads family proteins are the key regulators in TGF-β signaling pathways. In this work, tiger17 was found to increase phosphorylation of both Smad2 and -3 after its treatment for 18 h. Compared with Smad2 phosphorylation level without tiger17 treatment, Smad2 phosphorylation was increased by 2.57, 7.14, 12.29 or 13.14 times after 2.5, 5, 10 and 20 μg/ml tiger17 treatment, respectively (Fig. 4A and B); The corresponding Smad3 phosphorylation was increased by 0.4, 0.91, 1.07 or 1.63 times, respectively (Fig. 4A and C). It had no significant effects on Smad7 expression (Fig. 4A and D).

### Effects of Tiger17 on MAPK Signaling Pathways

**Figure 5:**
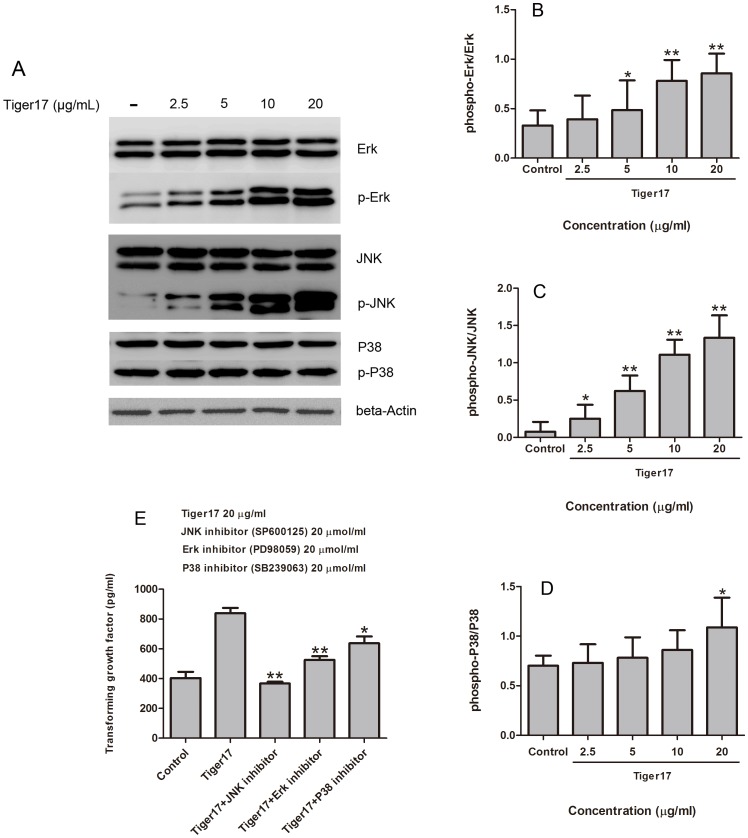
Effects of tiger17 on the MAPKs signal pathway. (A) Raw 264.7 cells were treated with the indicated concentrations of tiger17 (2.5, 5, 10, 20 μg/ml) or vehicle; Western blotted for total or phospho-JNK, Erk or P38 was performed. β-Actin was used as the internal control. (B, C, D) The band intensity of various inducer of MAPKs pathway = each phosphate-contents/homologous total-contents. (NIH, Image J). (E) After MAPKs pathway inhibitors were used, TGF-β1 expression was decreased, even the same way tiger17 treatment. Values are the mean ± SE of three independent experiments, *P<0.05, **P<0.01.

Many proofs have indicated that MAPK signaling pathways have crosstalk with TGF-β signaling pathways that commonly regulate TGF-β expression [20]. Tiger17’s effects on MAPK signaling pathways were assayed in RAW 264.7 macrophage cells by using Western immunoblot analysis. As shown in Fig. 5, tiger17 increased JNK, Erk and P38 phosphorylation in a concentration-dependent manner, especially for JNK and Erk phosphorylation. The Erk phosphorylation was increased ∼0.3, 0.63, 1.67 or 1.89 times by 2.5, 5, 10 and 20 μg/ml tiger17 treatment for 1 h, respectively (Fig. 5A and B). JNK phosphorylation level has been significantly increased by ∼2.13, 6.75, 12.88 or 15.75 times after 2.5, 5, 10 and 20 μg/ml tiger17 treatment, respectively (Fig. 5A and C). 5, 10 and 20 μg/ml tiger17 treatment increased P38 phosphorylation by ∼0.12, 0.23 and 0.56 times, respectively (Fig. 5A and D). Inhibitors (25 μmol/ml) of MAPKs signal pathway, PD98059, SP600125 or SB239063, was added into well before tiger17 treatment, respectively. And then, TGF-β1 contents were determined by ELISA in the cell-free supernatant. As shown in Fig. 5E, it was clearly displayed that TGF-β1 expression was decreased after any inhibitor treatment. Down-regulation of TGF-β1 was the most obvious after JNK inhibitor (SP600125) treatment. After P38 inhibitor (SB239063) treatment, TGF-β1 expression was slightly down-regulated.

## Discussion

Impaired wound healing continues to be a major health problem that predisposes to infections, long-term morbidity and mortality, particularly in high risk patients who maybe consequently suffer from unhealing skin ulcer even amputation [6], [21], [22]. Therefore, developing new drugs and treatment methods will be eagerly expected. A designed peptide (tiger17) was demonstrated to contain excellent skin wound healing ability in this study. Cyclic tiger17 directly functioned in keratinocytes proliferation and migration (Fig. 1A and B), and stimulated RAW264.7 macrophages to produce cytokines TGF-β1 and IL-6 (Fig. 3A and B). Topical application of tiger17 significantly reduced the wound closure time in a full-thickness skin wound model (Fig. 2A and B). This indicated that topical application of tiger17 had a potent healing effect on full-thickness traumatic skin wound.

Wound repair is a complex process that can be roughly divided into three continuous and overlapping phases: inflammatory reaction, proliferative phase and tissue remodeling [23]. The proliferation and migration of epithelial cells are key events for the formation of epithelium [24]–[26]. During this process, keratinocytes migrate to cover the wound surface and fill the wound space, thereby contributing to wound re-epithelialization and healing [27]. Moreover, contraction of differentiated myofibroblasts in granulation tissue was intensively beneficial for wound healing [28], [29]. Tiger17 treatment markedly enhanced the migration of keratinocytes (Fig. 1B) and promoted the proliferation of keratinocytes (Fig. 1A). Its therapeutic potential on skin wound was evaluated in a mouse model of full-thickness skin wound. Topical application of tiger17 greatly accelerated full-thickness skin wound healing (Fig. 2A). It promoted the re-epithelialization of the wound skin (Fig. 2C). These results suggested that tiger17 enhanced the proliferation and migration of keratinocytes eventually causing faster neoepithelial tongue formation and accelerating cutaneous wound healing.

The healing process of the injured site is an extremely complex process involving numerous cell types as well as growth factors, cytokines, and extra-cellular matrix (ECM) components [30]. Among this many cell types, macrophages has very key effects on wound healing. Macrophages almost take part in every process during the wound healing, which can phagocytose pathogens and present antigens to T-cells [31], [32]. Moreover, macrophages can produce numerous cytokines to participate the wound healing procedure [33]–[35]. The secretion of both IL-6 and TGF-β1 was elevated in macrophage RAW264.7 cells by tiger17 treatment in a concentration-dependent manner (Fig. 3A and B). TGF-β1 elicits its cellular responses principally through the canonical Smads signal pathway [36]. For TGF-β1, receptor-activated Smads are Smad2 and Smad3, and inhibitor Smad is Smad7 [37], [38]. Tiger17 increased activation of both Smad2 and Smad3 in macrophages as expected (Fig. 4A, B and C).

In addition, immunohistochemical analysis indicated that tiger17 promoted macrophages recruiting to the margin of wound area (Fig. 3C). TGF-β1 expression around the wound area was also elevated by tiger17 treatment (Fig. 3E). These results suggested that tiger17 potently recruited macrophages to unwounded skin margin which near to neo-epidermis and granulation tissue, and agminated macrophages exerted their key roles on wound healing including the induction of TGF-β1 secretion, which possibly induces fibroblast-to-myofibroblast transition as described below.

Fibroblasts to myofibroblasts differentiation results in wound contraction and effectively drives wound healing, which is largely regulated by TGF-β1 [39]. Myofibroblasts express a highly characteristic protein, α-smooth muscle actin (α-SMA), which are responsible for wound contraction [40]. Tiger17 treatment promoted wound contraction as illustrated in Fig. 2A and C. Immunohistochemical analysis indicated that α-SMA expression was elevated by tiger17 treatment in the skin wound (Fig. 3G). The results suggested that tiger17 promoted macrophages recruitment, TGF-β1 expression and fibroblasts to myofibroblasts differentiation as mentioned above.

MAPKs have been demonstrated to take an important role in wound healing [20]. Results from western blot analysis indicated that tiger17 significantly increased the activation of JNK and Erk sub-group of MAPK signaling pathway and appeared to be a concentration-dependent manner (Fig. 5A). In order to further determine whether these kinases are involved in the process of tiger17 induced TGF-β1 secretion, we used specific kinase inhibitors to treat cells and tested the level of TGF-β1 in the supernatants through ELISA. Results indicated that the supernatants of the cells treated with JNK and Erk specific inhibitors showed a really significant reduced TGF-β1 level (Fig. 5E). This demonstrated that both JNK and Erk signaling pathways have been involved in Tiger17 stimulated TGF-β1 release and they may have a crosstalk and orchestrate in regulating TGF-β expression and wound healing process. According to these data obtained from cellular and molecular level, although tiger 17 exerted little effect on macrophages proliferation (Fig. 1A), it was manifested that tiger 17 obviously promoted macrophages recruitment to wound site (Fig. 3C), which suggested that the therapeutic effect of tiger 17 was related to macrophages recruitment, instead of proliferation.

Some growth factors, i.e EGF, have been clinically used to enhance wound healing of a variety of tissues. Besides their high cost for production, store and ship, growth factors may also exhibit the ‘tumor promotion’ like effect, which raises doubts on the clinical use. Therefore small molecules containing functions to promote endogenous wound healing agents production (such as TGF-β) might be a good selection for clinical application. The current small peptides containing only 11 amino acid residues might be a potential biomaterial or template for development of novel wound-healing agent.
